# A medical image segmentation method based on multi-dimensional statistical features

**DOI:** 10.3389/fnins.2022.1009581

**Published:** 2022-09-15

**Authors:** Yang Xu, Xianyu He, Guofeng Xu, Guanqiu Qi, Kun Yu, Li Yin, Pan Yang, Yuehui Yin, Hao Chen

**Affiliations:** ^1^College of Automation, Chongqing University of Posts and Telecommunications, Chongqing, China; ^2^Department of Computer Information Systems, Buffalo State College, Buffalo, NY, United States; ^3^Chongqing Key Laboratory of Translational Research of Cancer Metastasis and Individualized Treatment, Chongqing University Cancer Hospital, Chongqing, China; ^4^Department of Cardiovascular Surgery, Chongqing General Hospital, University of Chinese Academy of Sciences, Chongqing, China; ^5^Department of Emergency, The Second Affiliated Hospital of Chongqing Medical University, Chongqing, China; ^6^Department of Cardiology, The Second Affiliated Hospital of Chongqing Medical University, Chongqing, China

**Keywords:** medical image segmentation, deep learning, convolutional neural network, transformer, neural network

## Abstract

Medical image segmentation has important auxiliary significance for clinical diagnosis and treatment. Most of existing medical image segmentation solutions adopt convolutional neural networks (CNNs). Althought these existing solutions can achieve good image segmentation performance, CNNs focus on local information and ignore global image information. Since Transformer can encode the whole image, it has good global modeling ability and is effective for the extraction of global information. Therefore, this paper proposes a hybrid feature extraction network, into which CNNs and Transformer are integrated to utilize their advantages in feature extraction. To enhance low-dimensional texture features, this paper also proposes a multi-dimensional statistical feature extraction module to fully fuse the features extracted by CNNs and Transformer and enhance the segmentation performance of medical images. The experimental results confirm that the proposed method achieves better results in brain tumor segmentation and ventricle segmentation than state-of-the-art solutions.

## 1. Background

Medical image segmentation is not only an important step in medical image analysis, but also an indispensable part of computer-aided diagnosis and pathology research. With the continuous development of computer vision in recent years, convolutional neural networks (CNNs), especially fully convolutional networks (FCNs), have made breakthroughs in the applications of medical image segmentation. For example, they have been applied to brain Magnetic Resonance Imaging (MRI) (Li et al., 2021), multi-organ segmentation, and cardiac ventricle (Moeskops et al., 2016; Hesamian et al., 2019). FCNs enable end-to-end image semantic segmentation and have evolved many variants during development, U-Net (Ronneberger et al., 2015), V-Net (Milletari et al., 2016), 3D U-Net (Çiçek et al., 2016), Res-UNet (Xiao et al., 2018), density-unet (Li et al., 2018), Y-Net (Mehta et al., 2018), etc. have been specially proposed for image and volume segmentation according to various medical imaging modalities. Existing CNN-based methods have good image segmentation performance. Due to the limitation of convolution kernel size, each convolution kernel only focuses on local information. Therefore, it is difficult for these existing methods to generate any long-distance dependencies when performing image segmentation tasks. The ability to construct global contextual information is crucial for intensive prediction tasks during medical image segmentation.

To effectively address the issues on global contextual information, Transformer (Vaswani et al., 2017; Dosovitskiy et al., 2020) was proposed to handle the issues in sequence-to-sequence prediction. It uses a completely attention-based encoder-decoder architecture, which is completely different from CNN-based methods. A one-dimensional sequence is taken as input, so Transformer has a powerful modeling ability, not only in constructing global context information. The powerful capability, works well for downstream tasks in the case of large-scale pre-training.

Transformer has been widely used in medical image segmentation, but it only focuses on building global context information at all stages. Therefore, its ability to obtain local information is weakened, and the lack of detailed location information encoding reduces the distinguishability between background and target. Various CNN architectures such as U-Net provide a way to extract low-level visual information, which can well compensate for the spatial details of Transformer's local information.

Therefore, considering the above-mentioned advantages, some studies integrated CNNs and Transformer. For example, TransUNet (Chen et al., 2021), first used CNNs to extract local features, and then applied Transformer to global context modeling. This architecture not only establishes a self-attention mechanism, but also reduces the loss of local feature resolution brought by Transformer, making it have better image segmentation accuracy. However, TransUNet is only a simple integration of CNNs and Transformer, and there are some shortcomings in practical applications.

The low-dimensional image texture features mainly include structural features and statistical features. The image information contained in these features plays an important role in semantic segmentation. Chen et al. (2018) proposed the DeepLabv3+ model by adding an encoder to the DeepLabv3 (Chen et al., 2017) model to achieve the extraction and fusion of both shallow and deep image features. Li et al. (2020) proposed an edge preservation module to enhance low-dimensional edge features, effectively improving the performance of semantic segmentation. However, the above methods are all applied to shallow features or low-dimensional edge features. Although low-dimensional statistical features play an importance role in grasping global image features, only a small percent of existing solutions try to analyze them.

Therefore, this paper proposes a hybrid feature extraction network based on CNNs and Transformer. The proposed network can not only utilize the Transformer's ability to construct global contextual information, but can also use the CNN's ability to capture local information. Additionally, in order to use the statistical image features, this paper designs a multi-scale statistical feature extraction module to extract statistical image features to improve segmentation performance.

## 2. Related work

### 2.1. Semantic segmentation network

In the past few years, CNNs have been used as the main framework for various computer vision tasks, especially in semantic segmentation. The mainstream medical image segmentation methods use the encoder-decoder structured FCN and U-Net. U-Net++ (Zhou et al., 2018) designs more dense skip connections based on U-Net. Res-UNet (Xiao et al., 2018) introduces a residual module in ResNet (He et al., 2016), and designs a deeper network for feature extraction.

In the past 2 years, Vision Transformer (ViT) (Dosovitskiy et al., 2020) has demonstrated its powerful modeling capability in computer vision tasks. ViT splits the source image into patches and uses these patches to perform self-attention operations. The Swin Transformer (Liu et al., 2021) uses the shift idea to calculate the attention of different windows and layer the corresponding feature maps. MedT (Valanarasu et al., 2021) improves gated self-attention and applies Transformer to medical image segmentation.

Some recent solutions try to use the advantages of CNN and Transformer by integrating the two architectures as a new backbone network. The CMT (Guo et al., 2022) block consists of a depthwise convolution-based local perception unit and a light-weight transformer module. CoAtNet (Dai et al., 2021) fuses the two frameworks based on MBConv and relative self-attention. TransUNet (Chen et al., 2021) first fuses the U-shape structure of Transformer and U-Net and applies Transformer to medical image segmentation.

### 2.2. Statistical features

Statistical features as low-dimensional texture features play a key role in improving semantic segmentation performance. Many existing solutions exploit the texture information of statistical features. Simonyan et al. (2013) applied Fisher vector layers to enhance features using handcrafting. Wang et al. (2016) first proposed learnable histograms for semantic segmentation and object detection. Zhu et al. (2021) proposed a texture enhancement module and a pyramid texture extraction module to extract image texture features for the enhancement of semantic segmentation performance.

## 3. Method

### 3.1. Semantic segmentation network

**Figure 1:**
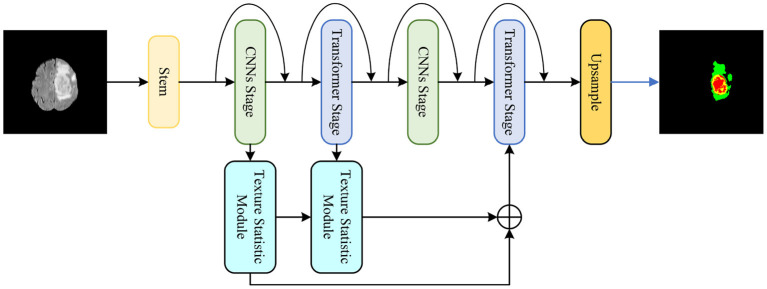
The proposed medical image segmentation method based on multi-dimensional statistical features.

A medical image segmentation method is proposed based on multi-dimensional statistical features as shown in Figure 1. This method integrates CNNs and Transformer into the feature extraction network, and designs a texture statistics extraction module (TSEM) for the extraction and fusion of multi-dimensional statistical features.

### 3.2. Hybrid network

**Figure 2:**
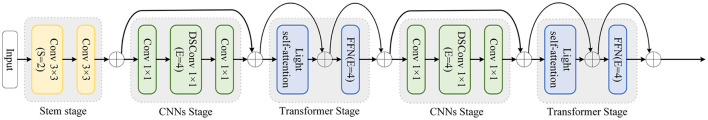
The proposed hybrid network consisting of CNNs stages and Transformer stage.

The proposed hybrid feature extraction network aims to utilize the advantages of CNNs and Transformer to achieve more accurate segmentation tasks. As shown in Figure 2, the proposed hybrid network is divided into five stages.

Stem is the first stage. CNNs and Transformer alternate in the remaining four stages. At the beginning of each stage, downsampling is applied to decrease feature map size and increase the number of channels. Additionally, the proposed network refers to the residual connection of ResNet and performs shortcuts at each stage.

Specifically, stem as the first stage contains two layers of simple 3 × 3 convolution. CNNs stage is the second stage, because the feature map is too large at this moment and not suitable for using Transformer in global feature extraction. The CNNs stage uses a Depthwise Separable Convolution block (DSConv) (Howard et al., 2017) to reduce the amount and size of model parameters. There is a 1 × 1 convolution layer before and after DSConv to change the feature map size and the number of channels. The third stage is the Transformer stage, which extracts global features after CNNs. The proposed network adopts a lightweight multi-head self-attention.

In the original self-attention module, the input *X* ∈ ℝ^*C*×*H*×*W*^ is linearized to query $Q\in {\mathbb{R}}^{n\times d_{k}}$, key $K\in {\mathbb{R}}^{n\times d_{k}}$, and value $V\in {\mathbb{R}}^{n\times d_{v}}$, where *n* = *H* × *W* is the number of patches, *d*, *d*_*k*_, *d*_*v*_ represent input, key, and value's dimension. The self-attention output is obtained by the following formula.


(1) $$\begin{array}{l} Atten\;(Q,K,V)=\text{Soft max }\left(\frac{QK^{T}}{\sqrt{d_{k}}}\right)\;V \end{array}$$

In order to reduce the overhead, the proposed network uses a *k*×*k* depthwise convolution with a stride of k to reduce the dimensions of *K*, *V*, ie $K^{\prime }=DSVConv\left(K\right)\in {\mathbb{R}}^{\frac{n}{k^{2}}\times d_{k}}$ and $V^{\prime }=DSVConv\left(V\right)\in {\mathbb{R}}^{\frac{n}{k^{2}}\times d_{v}}$, so the lightweight attention output is obtained by the following formula.


(2) $$\begin{array}{l} Atten\;(Q,K,V)=\text{Soft max }\left(\frac{Q{K^{\prime }}^{T}}{\sqrt{d_{k}}}\right)\;V^{\prime } \end{array}$$

The CNNs and Transformer operations in the second and third stages are repeated in the subsequent fourth and fifth stages. Additionally, each stage is repeated L times. Stages 1 to 5 of the proposed network are were repeated 2, 2, 4, 2, and 8 times, respectively.

### 3.3. Texture statistics extraction module

**Figure 3:**
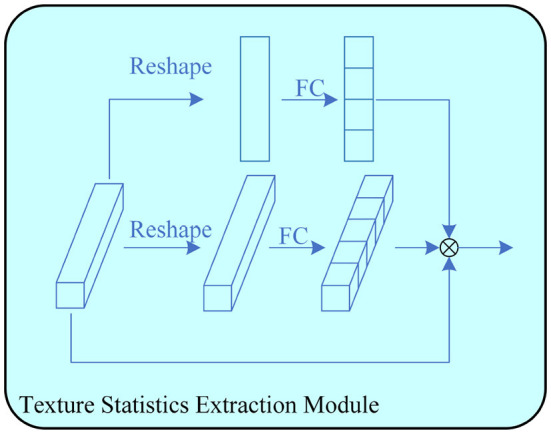
The proposed Texture Statistics Extraction Module. It is used to extract statistics at different stages.

The image texture information contains local structural features and global statistical properties. For poorly visualized images, the global statistical features are more suitable for segmentation. To effectively utilize statistical image features, a texture statistics extraction module (TSEM) is proposed. TSEM extracts statistical image features by encoding feature maps, as shown in Figure 3.

Given an input feature map *X* ∈ ℝ^*C*×*H*×*W*^, the input is divided into three branches for multi-scale feature encoding. One branch is first processed by global average pooling to obtain channel average features, and then multiplied with the input feature map *X* ∈ ℝ^*C*×1×1^ to obtain the final output feature map. Another branch first average pooling on one channel to obtain the feature map *X* ∈ ℝ^1×*H*×*W*^, and then multiplies it with the input feature map *X* ∈ ℝ^*C*×*H*×*W*^ to obtain the output feature map. The last two input feature maps are multiplied to obtain the output feature map of this module.

### 3.4. Loss function

To achieve the end-to-end training effect, a fusion loss function *L*_*fusion*_ is used to optimize the proposed method in the training process, training segmentation prediction and ground truth (GT). The loss function uses BCEDiceLoss, which is composed of binary cross entropy loss (BCELoss) and dice loss. The formula is given as follows:


(3) $$\begin{array}{l} L_{fusion}=\sum (0.5*\left(-y\log\left(ŷ\right)-\left(1-y\right)\log\left(1-ŷ\right)\right)\\ \;+\left(1-\frac{2|y\cap ŷ|}{\left|y|+|ŷ\right|}\right)) \end{array}$$

Where *y* represents GT and ŷ represents the network prediction result.

## 4. Experiments

### 4.1. Datasets

To verify the effectiveness of the proposed method, BraTS2018 (Menze et al., 2014; Bakas et al., 2017, 2018) and the cardiac segmentation dataset in the medical segmentation (Antonelli et al., 2022) decathlon are used as training and testing datasets in the experiments. The BraTS2018 dataset has 285 annotated brain tumor magnetic resonance imaging (MRI) cases, and each case has four different modalities, namely Flair, T1, T1ce, and T2. This dataset needs to segment three different brain tumor regions, which are Whole Tumor (WT), Tumor Core (TC), Ehance Tumor (ET). The decathlon cardiac segmentation dataset contains 20 annotated mono-modal MRI cases, and this dataset requires the segmentation of the left atrium.

### 4.2. Experimental details

The model frameworks in this paper are all implemented based on Pytorch. The image size and batch size of the input BraTS2018 dataset are 240*240 and 8, respectively. The image size and batch size of the input cardiac dataset are 320*320 and 8, respectively. Four Tesla P100 GPUs were used in training. Adam (Kingma and Ba, 2014) is the optimizer used in this paper, and all parameters are set as default. The initial learning rate and weight decay for model training are 1e-3 and 1e-5, respectively.

### 4.3. Comparative experiments

To verify the efficiency of the proposed model framework, three most common metrics used in medical image segmentation, IoU score, Dice score and Hausdorff score (HD) are used. The corresponding formulas are given:


(4) $$\begin{array}{l} IoU=\frac{Y\cap Ŷ}{Y\cup Ŷ} \end{array}$$

(5) $$\begin{array}{l} Dice=\frac{2|Y\cap Ŷ|}{\left|Y|+|Ŷ\right|} \end{array}$$

Where *Y* represents GT and Ŷ represents the network prediction result.


(6) $$\begin{array}{l} H\left(A,B\right)=\max\left(\underset{a\in A}{\max}\;\{\underset{b\in B}{\min}\|a-b\|\},\;\underset{b\in B}{\max}\left\{\underset{a\in A}{\min}\|b-a\|\right\}\right) \end{array}$$

Where *A* = {*a*_1_, *a*_2_, ..., *a*_*p*_}, *B* = {*b*_1_, *b*_2_, ..., *b*_*q*_} represents the pixels of the prediction result and GT. ||·|| represents the norm between *A* and *B*.

**Figure 4:**
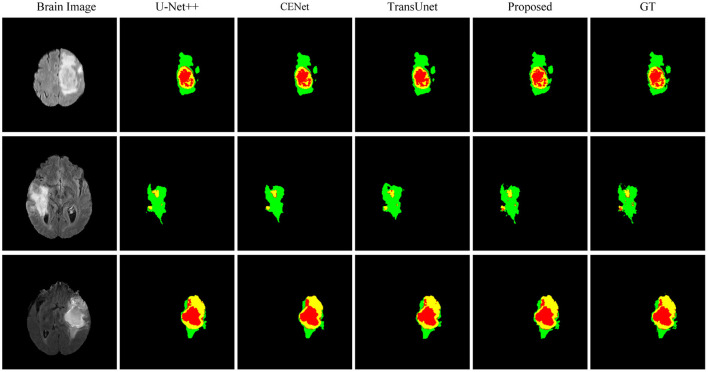
Comparison of the proposed method and other state-of-the-art methods on BraTS2018.

**Figure 5:**
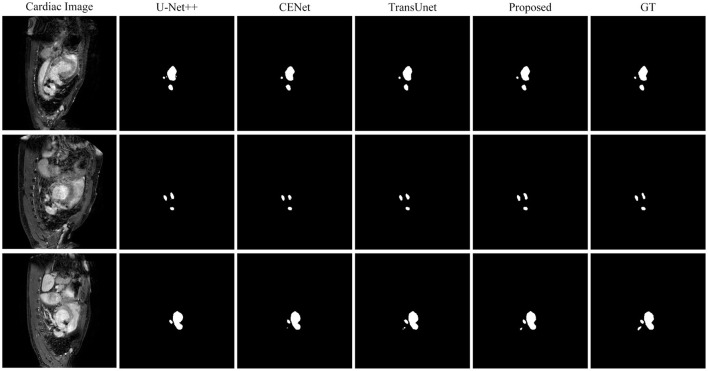
Comparison of the proposed method and other state-of-the-art methods on the Cardiac Dataset.

This paper conducts comparative experiments with state-of-the-art image segmentation frameworks on the BraTS2018 and cardiac segmentation datasets. These frameworks include 2D CNN, 3D CNN segmentation frameworks (Ronneberger et al., 2015; Myronenko, 2018; Zhou et al., 2018; Gu et al., 2019; Zhang et al., 2020) and partial Transformer segmentation framework (Chen et al., 2021). The corresponding experimental results obtained by each method are shown in Tables 1, 2, and the visualized results are shown in Figures 4, 5. The number of parameters and computation cost are compared, as shown in Table 3.

**Table 1:** Comparison of segmentation metrics on BraTS2018.

**BraTS2018**	**WT**		**TC**		**ET**		**Average**	
	**Dice**	**HD**	**Dice**	**HD**	**Dice**	**HD**	**Dice**	**HD**
Myronenko	90.40	4.483	85.90	8.278	81.40	3.805	85.90	5.500
U-Net++	88.96	5.327	84.65	8.535	79.49	4.285	84.36	6.049
CENet	89.53	5.271	84.31	8.493	79.95	4.379	84.60	6.193
D. Zhang	89.60	5.733	82.40	9.270	78.20	3.567	83.40	6.190
TransUNet	90.25	**4.390**	**87.19**	5.539	80.41	3.731	85.95	4.553
**Proposed**	**90.45**	4.923	86.96	**5.327**	**81.53**	**3.279**	**86.31**	**4.510**

Bold font represents the best result.

**Table 2:** Comparison of segmentation metrics on medical segmentation decathlon.

**Cardiac Dataset**	**IoU**	**Dice**	**HD**
U-Net	90.07	93.86	1.7414
U-Net++	90.55	94.38	1.7197
CENet	90.23	94.17	1.7682
TransUNet	90.67	94.54	1.7300
**Proposed**	**91.30**	**94.86**	**1.6772**

Bold font represents the best result.

**Table 3:** Comparison of the model size and flops cost.

**Model**	**Input size**	**Parameter(M)**	**FLOPS(G)**
U-Net	3, 224, 224	39.40	55.84
U-Net++	3, 224, 224	9.34	34.65
TransUNet	3, 224, 224	105.32	38.52
MedT	3, 224, 224	**1.60**	21.24
**Proposed**	3, 224, 224	37.25	**15.24**

Bold font represents the best result.

According to the comparison results, the proposed segmentation framework obtains better scores and achieves a more significant performance improvement compared with state-of-the-art segmentation models. The proposed segmentation model achieves an average Dice of 86.31% on the BraTS2018 dataset and an average Dice of 94.86% on the medical segmentation decathlon, which are better than other state-of-the-art segmentation models.

According to the visualized results shown in Figure 4, the proposed method significantly improves the refinement of tumor and its texture features by using TSEM. Compared with other state-of-the-art, the model developed based on the integration of CNNs and Transformer has achieved better results in the context feature extraction and statistical feature fusion, and provides a reference for medical image segmentation of brain tumors and hearts. According to Table 3, the proposed method also has the lowest flops.

### 4.4. Ablation experiments

**Figure 6:**
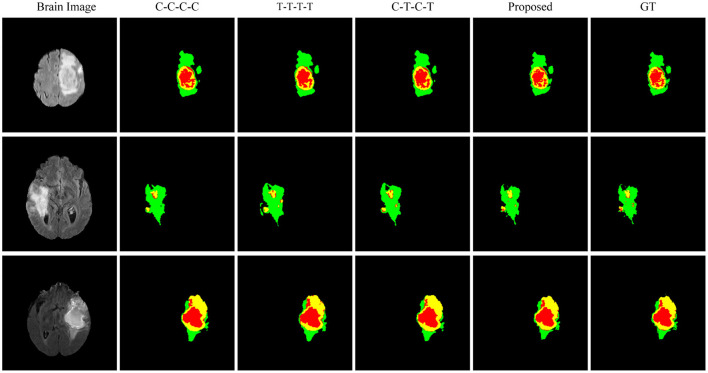
Visual results of ablation experiments on BraTS2018.

**Figure 7:**
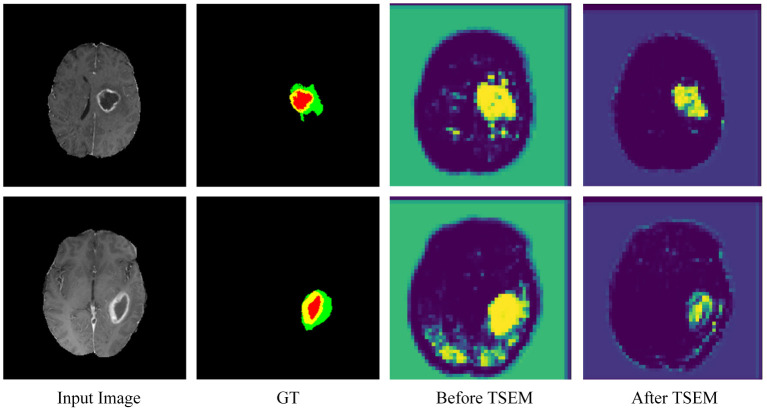
Performance comparison before and after adding TSEM.

In order to further verify the importance and practical contribution of the backbone network used in this paper and the designed modules, the relevant ablation experiments are carried out. The index comparison of ablation experiments is shown in Table 4, and the experimental results are shown in Figures 6, 7.

**Table 4:** Ablation experiment results on BraTS2018.

**BraTS2018**	**WT**		**TC**		**ET**		**Average**	
	**Dice**	**HD**	**Dice**	**HD**	**Dice**	**HD**	**Dice**	**HD**
C-C-C-C	88.31	5.322	86.32	5.531	80.68	4.293	85.10	5.049
T-T-T-T	88.15	5.514	86.19	6.681	80.64	4.450	84.99	5.548
C-T-C-T	89.04	5.357	86.91	5.554	80.91	3.315	85.32	4.742
**Proposed**	**90.45**	**4.923**	**86.96**	**5.327**	**81.53**	**3.279**	**86.31**	**4.510**

Bold font represents the best result.

This paper uses a fully convolutional layer as the Baseline for segmentation, and then replaces the backbone network blocks one by one for experiments. The experiments cover the full convolution network of C-C-C-C, the full transformer network of T-T-T-T, the hybrid network of C-T-C-T, and the TSEM is finally. The corresponding indicator values are shown in Table 4. The proposed module can improve the segmentation performance of baseline to a certain extent. After adding TSEM to the baseline, the corresponding improvement is the most obvious.

According to Table 4, the average Dice of the full Transformer is slightly lower than the result of the full CNN. The C-T-C-T result of the integration of CNNs and Transformer is significantly improved, confirming the effectiveness of the proposed hybrid network. After adding TSEM, the corresponding performance is further improved, the Dice of WT is increased by 1.41%, and the average Dice is increased by 0.99%.

Figure 6 shows the visualized brain tumor segmentation results obtained by each method in ablation experiments. After the backbone network becomes a hybrid network, the segmentation performance is further improved. After adding the texture statistics extraction module, the brain tumor edges after segmentation are significantly better, and the involved edges regions are closer to the actual situation compared with the segmentation result obtained by the hybrid network.

To further verify the role of TSEM, an intermediate experimental procedure is added. As shown in Figure 7, the area of interest in the feature map is concentrated and accurate after adding TSEM. Before adding TSEM, the feature map is mainly concentrated in the segmented area. Therefore, the proposed TSEM is conducive for the network to paying more attention to the segmented area and can effectively improve segmentation results.

## 5. Conclusion

This paper proposes a medical image segmentation method based on multi-dimensional statistical features. It consists of a hybrid feature extraction network and a multi-dimensional statistical feature extraction module. The hybrid feature extraction network is composed by CNNs and Transformer, and the lightweight processing is adopted to adapt to practical application scenarios. The multi-dimensional statistical feature extraction module is used to strengthen low-dimensional image texture features and enhance medical image segmentation performance. Experimental results show that the proposed medical image segmentation method achieves excellent results on brain tumor and heart segmentations.

## Data availability statement

Publicly available datasets were analyzed in this study. This data can be found here: http://medicaldecathlon.com/; https://wiki.cancerimagingarchive.net/pages/viewpage.action?pageId=37224922.

## Author contributions

YX, XH, and GX: conceptualization, data curation, and visualization. XH and GQ: methodology and writing—original draft preparation. YX, XH, and GQ: software. KY, LY, and YY: validation. LY and HC: formal analysis. YX: investigation. YY and HC: resources. GQ, HC, LY, and YY: writing—review and editing. HC, LY, and YY: supervision. YX and HC: project administration. HC: funding acquisition. All authors have read and agreed to the published version of the manuscript.

## Funding

This research was sponsored by the China Postdoctoral Science Foundation (2020M670111ZX), Chongqing medical scientific research project (Joint project of Chongqing Health Commission and Science and Technology Bureau, 2020GDRC019 and 2022MSXM184), Natural Science Foundation of Chongqing (cstc2020jcyj-bshX0068), and Special Fund for Young and Middle-aged Medical Top Talents of Chongqing (ZQNYXGDRCGZS2019005).

## Conflict of interest

The authors declare that the research was conducted in the absence of any commercial or financial relationships that could be construed as a potential conflict of interest.

## Publisher's note

All claims expressed in this article are solely those of the authors and do not necessarily represent those of their affiliated organizations, or those of the publisher, the editors and the reviewers. Any product that may be evaluated in this article, or claim that may be made by its manufacturer, is not guaranteed or endorsed by the publisher.
